# Blood levels of pro-inflammatory and anti-inflammatory cytokines during an oral glucose tolerance test in patients with symptoms suggesting reactive hypoglycemia

**DOI:** 10.1590/1414-431X20165195

**Published:** 2016-07-11

**Authors:** W. Eik, S.S. Marcon, T. Krupek, I.T.S. Previdelli, O.C.N. Pereira, M.A.R.C.P. Silva, R.B. Bazotte

**Affiliations:** 1 Disciplina de Endocrinologia, Departamento de Medicina, Universidade Estadual de Maringá, Maringá, PR, Brasil; 2 Programa de Pós Graduação em Ciências da Saúde, Centro de Ciências da Saúde, Universidade Estadual de Maringá, Maringá, PR, Brasil; 3 Departamento de Enfermagem, Universidade Estadual de Maringá, Maringá, PR, Brasil; 4 Departamento de Farmacologia e Terapêutica, Universidade Estadual de Maringá, Maringá, PR, Brasil; 5 Departamento de Estatística, Universidade Estadual de Maringá, Maringá, PR, Brasil; 6 Programa de Pós Graduação em Bioestatística, Universidade Estadual de Maringá, Maringá, PR, Brasil

**Keywords:** Pro-inflammatory cytokines, Anti-inflammatory cytokines, Glucose tolerance test, Inflammation, Postprandial glycemia

## Abstract

We evaluated the impact of postprandial glycemia on blood levels of pro-inflammatory and anti-inflammatory cytokines during an oral glucose tolerance test in non-diabetic patients with symptoms suggesting reactive hypoglycemia. Eleven patients with clinical symptoms suggesting reactive hypoglycemia received an oral glucose solution (75 g) Blood was collected at 0 (baseline), 30, 60, 120 and 180 min after glucose ingestion and the plasma concentrations of interferon-α (IFN-α), interferon-γ (IFN-γ), interleukin-1 receptor antagonist (IL-1RA), interleukin 2 (IL-2), interleukin-2 receptor (IL-2R), interleukin 4 (IL-4), interleukin 6 (IL-6), interleukin 8 (IL-8), interleukin 10 (IL-10), interleukin-12 (IL-12), interleukin 13 (IL-13), interleukin 15 (IL-15), interleukin 17 (IL-17), IFN-γ inducible protein 10 (IP-10), monocyte chemotactic protein 1 (MCP1), monokine induced by IFN-γ (MIG), macrophage inflammatory protein-1α (MIP-1α), interleukin-1β (IL-1β), colony stimulating factor (G-CSF), granulocyte-macrophage CSF (GM-CSF), basic fibroblast growth factor (FGF-basic), eotaxin, tumor necrosis factor α (TNFα), epidermal growth factor (EGF), hepatocyte growth factor (HGF), vascular endothelial growth factor (VEGF), macrophage inflammatory protein-1α (MIP-1α), and 1β (MIP-1β) were evaluated. Overall, glycemic levels increased, reached its maximum at 30 min (phase 1), returned to baseline levels at 120 min (phase 2), followed by a mild hypoglycemia at 180 min (phase 3). During phase 1, cytokine blood levels were maintained. However, we observed a synchronous fall (P<0.05) in the concentrations of pro-inflammatory (IL-15, IL-17, MCP-1) and anti-inflammatory cytokines (FGF-basic, IL-13, IL-1RA) during phase 2. Furthermore, a simultaneous rise (P<0.05) of pro-inflammatory (IL-2, IL-5, IL-17) and anti-inflammatory cytokines (IL-4, IL-1RA, IL-2R, IL-13, FGF-basic) occurred during phase 3. Thus, mild acute hypoglycemia but not a physiological increase of glycemia was associated with increased blood levels of anti-inflammatory and pro-inflammatory cytokines.

## Introduction

Cytokines are small proteins with multiple biological properties (1–6) including growth factors (7), interferons (8), interleukins (9), chemokines (10) and the tumor necrosis factor family (11).

Early studies on cytokines focused on inflammation, but today it is recognized that cytokines mediate a multiplicity of biological processes. For example, interleukin 4, initially described as β cell growth factor, also showed anti-inflammatory properties, and leptin, initially described as a suppressor of appetite, also works as a pro-inflammatory cytokine (8).

On one hand, cytokines mediate a number of biological processes (8), and, on the other hand, a number of biological processes, including blood glucose changes (12,13), influence cytokine blood levels. Moreover, in spite of the fact that acute hyperglycemia (13) and hypoglycemia (14,15) are associated with increased blood levels of pro-inflammatory cytokines in non-diabetic subjects, to our knowledge, there are no data that correlates anti-inflammatory cytokines with those conditions.

In this context, we aimed to evaluate the effect of the acute increase and decrease of glycemia on circulating pro-inflammatory and anti-inflammatory cytokines levels by assessing patients with clinical symptoms suggesting reactive hypoglycemia. The reason for selecting these specific individuals was the possibility to obtain a glycemic curve with a hyperglycemic followed by a hypoglycemic phase.

## Material and Methods

The study was approved by the Ethics Committee of the Universidade Estadual de Maringá (Protocol CAAE #0164.0.093.000-11) and written informed consent to participate in this investigation was obtained from each patient.

Patients seeking treatment for symptoms suggestive of reactive hypoglycemia at the first author’s (W. E-F) private clinic were recruited. Eligibility criteria were: age between 18 to 59 years, and agreement to participate in all of the activities proposed in the study. Exclusion criteria were: any type of diabetes, pregnancy or risk of pregnancy, breastfeeding, any disease or condition that could influence the investigation including cardiovascular, intestinal, liver, kidney and infectious diseases, body mass index >30 kg/m^2^, medical history of stroke, smoking, alcoholism or consumption of any illegal or medicinal drugs, elective surgery coinciding with the period of participation in the study, not understanding the study protocol, previous participation in other trials within 6 months prior to entry into this study.

A total of 11 patients were included in the study. On the day of the oral glucose tolerance test (OGTT), the patients (after 10-h overnight fast) received oral glucose solution (75 g), and venous blood samples were collected from the median cubital vein at 0 (baseline values), 30, 60, 120, and 180 min after glucose ingestion. From the blood sample collected at time 0, we measured glycated hemoglobin A1c, total cholesterol, triacylglycerol and C-peptide.

In addition, from plasma samples that were stored at -80°C, we measured plasma glucose and cytokines. ACTH, cortisol and insulin were also evaluated, as it is well established that their abnormal levels represent favorable conditions for hypoglycemia. Moreover, from the basal values (time 0) of glycemia and insulinemia, we calculated the homeostatic model assessment-estimated insulin resistance (HOMA-IR), according to the formula: fasting insulin (µU/L) × fasting glucose (nmol/L)/22.5.

Hypoglycemia diagnosis during the OGTT was based on Whipple's triad, i.e., glycemia <70 mg/dL, symptoms consistent with hypoglycemia and improvement or resolution of symptomatology after elevation of glycemia.

Blood glucose, glycated hemoglobin A1c, total cholesterol and triacylglycerol were determined as previously described (16). Quantitative measurement of cortisol, ACTH, C-peptide and insulin were done using chemiluminescence enzyme immunoassay kits for use on the Immulite® and Immulite® 1000 systems (Siemens, USA).

We evaluated the following pro-inflammatory cytokines: interleukin 1β (IL-1β), interleukin 2 (IL-2), IL 2 receptor (IL-2R), interleukin 5 (IL-5), interleukin 6 (IL-6), interleukin 8 (IL-8), interleukin 12 (IL-12), interleukin 15 (IL-15), interleukin 17 (IL-17), epidermal growth factor (EGF), vascular endothelial growth factor (VEGF), eotaxin, granulocyte-macrophage colony-stimulating factor (GM-CSF), interferon α (IFN-α), interferon γ (IFN-γ), monokine induced by IFN-γ (MIG), inducible protein 10 (IP-10), monocyte chemotactic protein 1 (MCP-1), macrophage inflammatory protein-1α (MIP-1-α), macrophage inflammatory protein-1β (MIP-1-ß), and tumor necrosis factor α (TNF-α).

Furthermore, we also evaluated the anti-inflammatory cytokines: interleukin 1 receptor antagonist (IL-1RA), interleukin 4 (IL-4), interleukin 6 (IL-6), interleukin 10 (IL-10), interleukin 13 (IL-13), basic fibroblast growth factor (FGF-basic), granulocyte-colony stimulating factor (G-CSF), and hepatocyte growth factor (HGF).

Cytokines were evaluated with the human cytokine magnetic plex panel (Invitrogen, USA) with the immunoassay Luminex Magpix® Plataform (USA).

A single database estimating a polynomial regression model of third degree to each variable response was obtained. Estimates generated from these adjustments were imputed into the database and the Hotteling *t*-test was used for paired comparisons between 0–30, 30–120, and 120–180 min. Results are reported as means±SD. P<0.05 indicated statistical significance.

## Results

The blood levels of glycated hemoglobin A1c, C-peptide, total cholesterol, and triacylglycerol were 5.3±0.2% (n=11), 1.9±0.2 mg/mL (n=10), 189.0±10.1 mg/dL (n=11) and 119.4±4.2 mg/dL (n=11), respectively.

In spite of the fact that all patients included in this study had clinical symptoms suggesting reactive hypoglycemia, during the OGTT only 2 patients showed glycemia <70 mg/dL and other signs of hypoglycemia (Table 1). We observed a glycemic curve with 3 phases: a first phase characterized by an elevation (P<0.05) of glycemia between 0 (baseline levels) and 30 min after glucose ingestion; a second phase characterized by a decrease (P<0.05) of glycemia and return to baseline levels (30–120 min), and a third phase (120–180 min) with an additional decrease of glycemia reaching values below (P<0.05) baseline levels (Table 2).

**Table:**
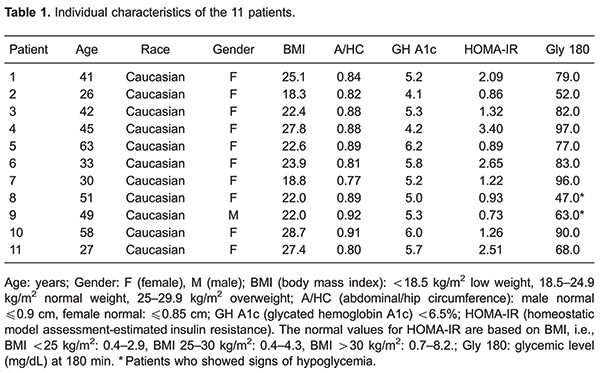

**Table:**
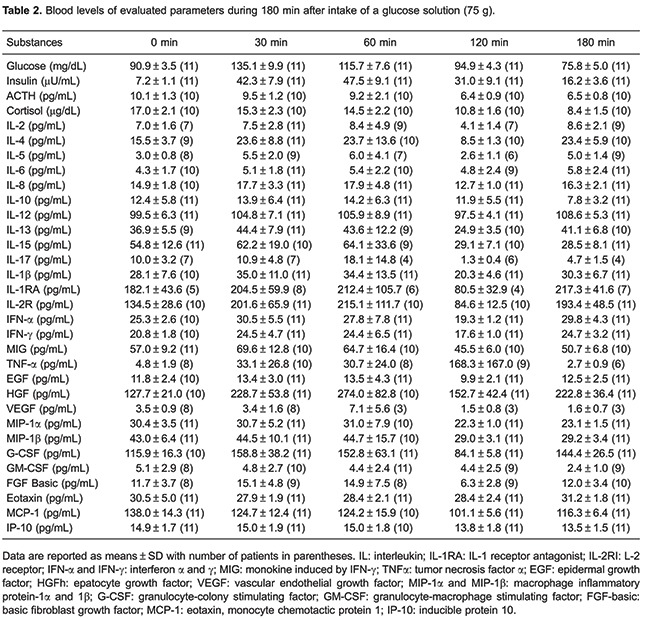

During the OGTT, the blood insulin levels increased (P<0.05) for 60 min after glucose ingestion and then progressively decreased until 180 min, while blood levels of ACTH and cortisol progressively decreased between 0 and 180 min. The HOMA-IR of all patients showed normal values (Table 1).

As shown in Table 3, during the first phase (0 to 30 min), blood levels of cytokines remained unchanged. In addition, part of the pro-inflammatory cytokines, i.e., IL-1β, INF-α, GM-CSF, EGF, VEGF, TNF-α, MIG, IL-8, IP-10, MIP-1-α, eotaxin, IFN-γ, IL-12, MIP-1ß, and the anti-inflammatory cytokines, i.e., IL-10, G-CSF, and HGF remained unchanged during OGTT. Moreover, IL-6, categorized as both a pro-inflammatory and anti-inflammatory cytokine, also remained unchanged during OGTT. However, we observed a synchronous fall (P<0.05) in the concentrations of pro-inflammatory (IL-15, IL-17, MCP-1) and anti-inflammatory cytokines (FGF-basic, IL-13, IL-1RA) during phase 2. Furthermore, a simultaneous rise (P<0.05) of pro-inflammatory (IL-2, IL-5, IL-17) and anti-inflammatory cytokines (IL-4, IL-1RA, IL-2R, IL-13, FGF-basic) occurred during phase 3.

**Table:**
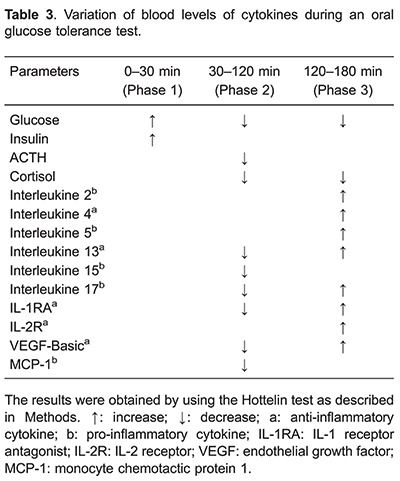

## Discussion

Considering that the majority of previous studies focused on circulating pro-inflammatory cytokines, the evaluation of anti-inflammatory cytokines represents an advance to understand how blood glucose levels influence blood cytokines levels.

The decrease in blood levels of the pro-inflammatory cytokines seen in this study could be attributed, at least partly, to the anti-inflammatory effect of insulin (17) that showed increased blood levels for 120 min after glucose ingestion. On the other hand, the rise in the blood levels of the pro-inflammatory cytokines could be attributed, partly at least, to the progressive decrease of cortisol blood levels during the OGTT.

We suggest that the simultaneous fall (30–120 min) and rise (120–180 min) of pro-inflammatory and anti-inflammatory cytokines during OGTT is indicative of an initial anti-inflammatory and subsequent pro-inflammatory balance. This hypothesis is in agreement with previously reported studies showing toll like receptors agonists inducing both pro-inflammatory and anti-inflammatory cytokines (8). Furthermore, other studies also demonstrated activation of pro-inflammatory and anti-inflammatory cytokines during neonatal sepsis (18), systemic infections in preterm infants (19) and type 1 diabetes (20).

Our results showing a predominance of anti-inflammatory effects associated with glycemia elevation in non-diabetic subjects contrasted with the results obtained by Esposito et al. (13). It must be considered, however, that those authors submitted their patients to non-physiological conditions including intravenous administration of glucose.

The novel findings of the present study were that mild acute hypoglycemia in non-diabetic subjects without insulin resistance (as inferred by HOMA-IR values) were associated with increased concentrations of pro-inflammatory and anti-inflammatory cytokines. This cytokine balance, as proposed by Elenkov et al. (6), could represent an important negative feedback mechanism, which protects the organism from an excessive inflammatory response and its consequences.

Finally, our results indicated that not only pro-inflammatory cytokines but also anti-inflammatory cytokines must be evaluated to obtain a complete picture about the impact of glycemic changes on the circulating levels of cytokines.
